# Fabrication and Specific Functionalisation of Carbon Fibers for Advanced Flexible Biosensors

**DOI:** 10.3389/fchem.2020.582490

**Published:** 2020-09-23

**Authors:** Zhang Wenrui, Meng Fanxing, Qin Yanan, Chen Fei, Yue Haitao, Zhang Minwei

**Affiliations:** College Life Science & Technology, Xinjiang University, Urumqi, China

**Keywords:** carbon fibers, fabrication, functionalization, compound material, flexible biosensor

## Abstract

This review aims at offering an up-to-date comprehensive summary of carbon fibers (CFs)-based composites, with the emphasis on smart assembly and purpose-driven specific functionalization for their critical applications associated with flexible sensors. We first give a brief introduction to CFs as a versatile building block for preparation of mutil-fountional materials and the current status of research studies on CFs. This is followed by addressing some crucial methods of preparation of CFs. We then summarize multiple possibilities of functionalising CFs, an evaluation of some key applications of CFs in the areas of flexible biosensors was also carried out.

## Introduction

**Figure 1:**
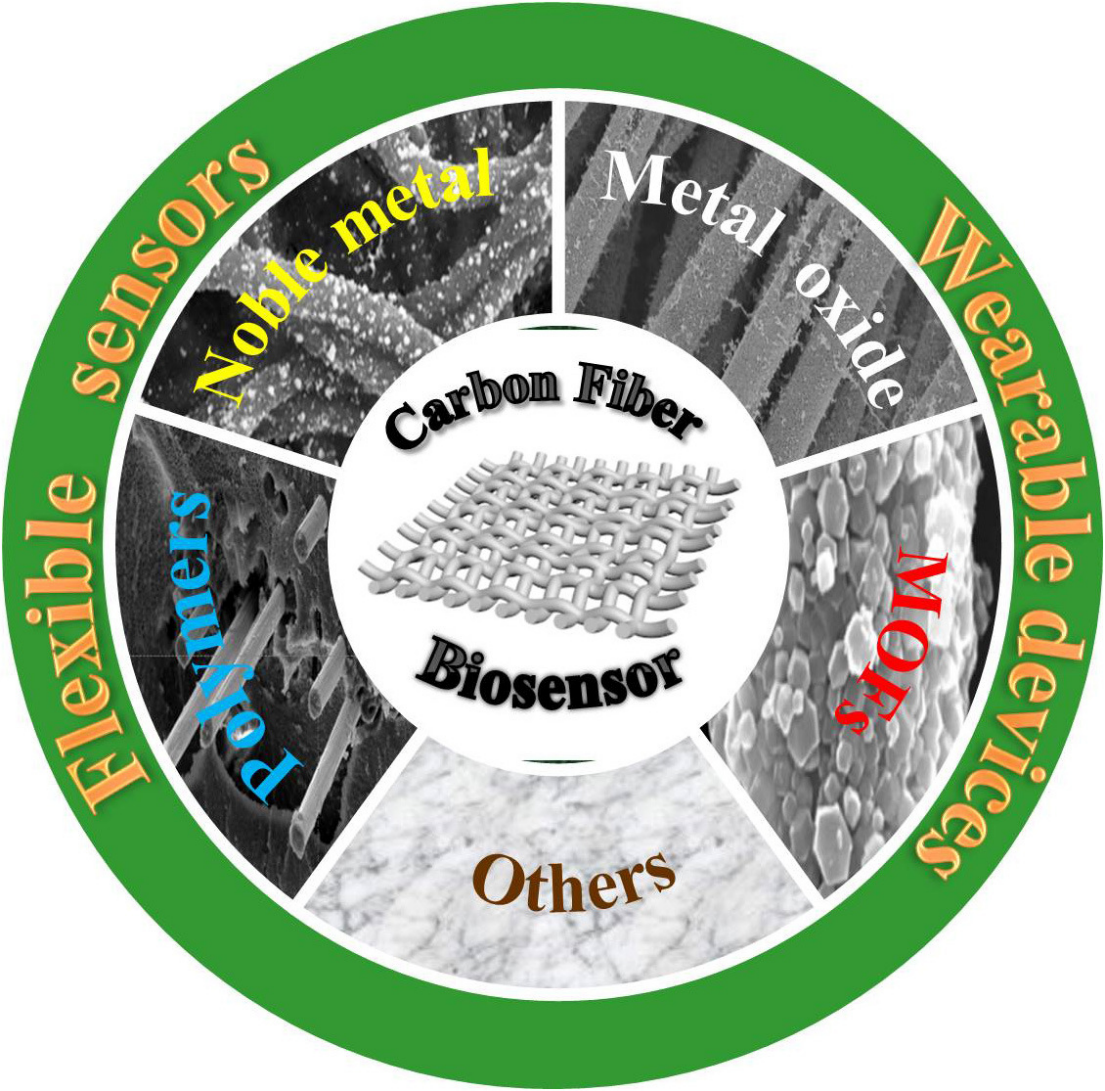
Functionalization of CFs and its application in biosensor.

Carbon fibers (CFs) being thin long filaments, contain more than 90 wt% of carbon and exhibit many outstanding properties such as high modulus (200–900 GPa), high compressive strength (up to 3 GPa), high tensile strength (2–7 GPa) (Nitilaksha et al., 2016; Meng et al., 2017), flexibility, and tunable electrochemical performance so that they can be widely used in various fields (Chen et al., 2020), such as aerospace, automobile, chemical industry, transportation, construction, sewage treatment and other fields. In addition, they can also serve as multifunctional hosts by a facile air-annealing process to get higher defective edge/plane sites, more oxygen-containing functional groups, which can load different electrochemical active substances such as noble metals, metal oxides, polymers, metal-organic frames (MOFs). As a result, they can be applied to fabricate electrochemical sensors with high sensitivity and flexibility as well as energy equipment (e.g., supercapacitors, batteries) with high energy/power density. The greatly improved performance has been found via combining pure CFs with the metal materials, metallic oxide materials, metallic sulfide materials, carbon materials and so on. For instance, the enhancement of performance was achieved by decorating CFs with cobalt oxide nanoparticles via solid-state mixing and thermal decomposition steps. As a result, the energy storage capacity of the capacitor is greatly enhanced. Moreover, CFs can be assembled into various structures applied in sensors, e.g., the electrode with porous structure can easily achieve the penetration of electrolyte and the diffusion of ions as well as the continuous conductive network could enable the rapid transfer of charge to active substances and metal ions (Zhang et al., 2019; Yang et al., 2020). CFs have excellent surface areas and can modify as many enzymes as possible, so thay are widely used as substrate materials for biosensors. In the review, we also summarize the current research progress of CFs-based biosensors and their applications in flexible and wearable biosensors. Figure 1 outlines the interest and focus of the present review.

## Preparation of CFs

It has been more than 100 years since the discovery of CFs. With the continuous updating of technology, the current preparation process of CFs has been very matured. Generally, CFs are prepared from synthetic fibers (precursor fibers) and different fiber raw materials need to use different production processes to prepare CFs. However, it is similar in the general process, which is stable precursor fiber pyrolysis and controlled stretching synthesis of CFs. At present, the main raw materials for the manufacture of CFs are polyacrylonitrile (PAN), pitch and rayon. In the actual production process, 90% of the CFs are produced from PAN-based precursors, and the remaining 10% are produced from precursors of asphalt or rayon (Bhatt and Goe, 2017). This is because compared with asphalt fiber and rayon fiber, polyacrylonitrile-based CFs have a higher strength, modulus and failure strain, and a higher yield rate. Carbon fiber (CF) is a kind of high strength material, which plays an important role in the fields of aerospace, automobile, chemical industry, general engineering, missile, nuclear, composite reinforcement and textile (Wazir and Kakakhel, 2009).

## CFs Classification Based on Precursors

### PAN-Based CFs

A PAN-based polymer is the best precursor for the production of CFs because of its tensile and compression properties and a carbon yield of up to 68% (Hamid et al., 2017). Wet spinning is used in the commercial production of CFs from most of the precursors of PAN-based polymers. However, wet spinning is gradually being replaced by the dry-jet-wet spinning, as it exhibits several advantages such as low adhesion between fibers and high specific surface area for improving the interlaminar shear strength of composite materials. Due to the isolation of extrusion expansion and epidermis solidification in dry-wet spinning, the formation mechanism of fiber has changed, so the phenomena such as cortical fracture and epidermis fold basically disappear in the process of wet spinning. The surface and internal defects of dry-wet spinning are reduced, and the density is increased. At the same time, dry-wet spinning also has the characteristics of high spinneret drawing, high spinning speed, easy to obtain high strength and high orientation fiber (Tian et al., 2017), so as to ensure the sufficient strength of CFs, which is the development direction of CFs production.

### Pitch-Based CFs

Pitch is produced by pyrolysing synthetic polymers. The molecular weight of pitch is in the range of 600–1,000 g/mol, and it contains aromatic groups. The diameter of pitch-based CFs ranges between 10 and 12 mm and the tensile strength and modulus of pitch-based CFs are−3 GPa and 960 GPa, respectively. Pitch is easy to be produced in large quantities and is attractive as a precursor for large-scale CFs manufacturing because the cost of pitch is significantly lower than that of other precursor fibers. Pitch-based precursors have other advantages that make them an attractive alternative, such as less energy required to convert aromatic graphitised materials and a lower proportion of hydrogen, nitrogen and other non-carbon elements (Wazir and Kakakhel, 2009; Yoshikawa et al., 2020).

### Rayon-Based CFs

Rayon is a manufactured fiber made up of cellulose extracted mainly from plants (cotton wool and pulp) (Chen X. et al., 2019). Cellulose is a promising raw material for CFs. Also, the cellulose precursor forms strong CFs by pyrolysis, which have high thermal conductivity, high purity, good mechanical toughness and low cost. The new production process requires that rayon fibers be carbonized into high modulus CFs filaments. In addition to the early low-strength fibers, this was followed by significantly increased yarns with high strength and high modulus of elasticity. However, due to the high cost of hot drawing, the production of these CFs has been delayed for many years, the CFs spinning process is low, and the properties of cellulose precursors are also delayed (Lee et al., 2017). Currently, only very few CFs are produced in this way.

### CFs Based on Other Precursors

In addition to the several widely used precursors mentioned above, other natural fibers such as silk and chitosan are also considered to be precursors for CFs manufacturing, which can reduce production costs, but can not provide strong mechanical properties. In addition, some linear and cyclic polymers have also been proved to be suitable for the preparation of CFs, but the results show that their carbon production is very low, thus hindering their further application (Khayyam et al., 2020).

### Spinning Classification

Generally speaking, the first step in the production process of CFs is to convert the powder or granular precursor into continuous fibers, that is, the spinning process (Lee et al., 2016). The frequently used spinning techniques can be classified as blow-spinning, electrostatic spinning, and centrifugal spinning depending on the force applied to the precursor solution/melt.

### Blow Spinning

**Figure 2:**
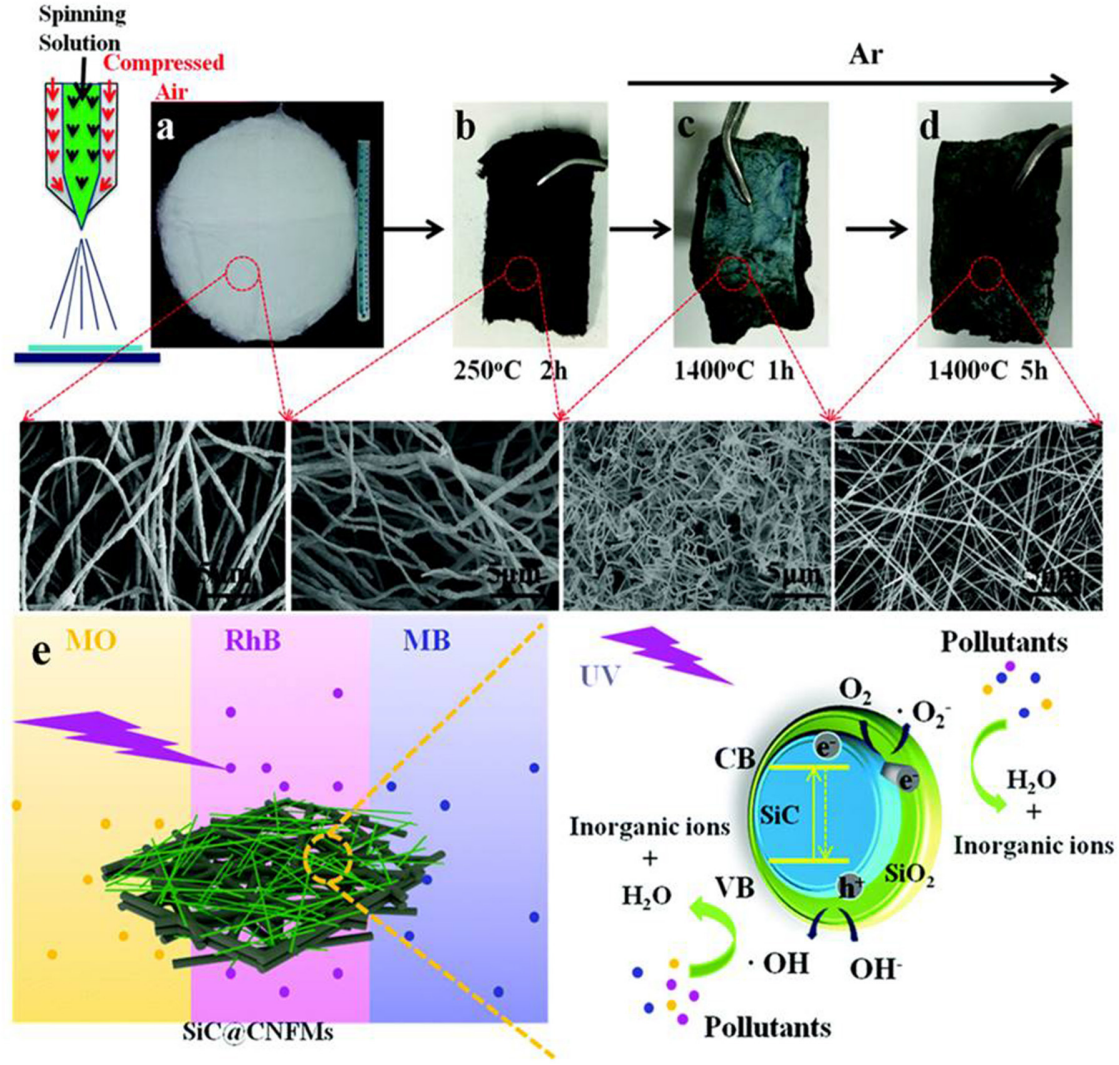
**(a–d)** Schematic illustration of the fabrication process of SiC@CNFMs. **(e)** Photocatalytic mechanism of the SiC@CNFMs-5. Reproduced with permission from Chen Y. et al. (2019).

Blow-spinning is a promising method for producing micro-/nano-fibers in large-scale production processes by using high-speed air. The spinning dope is loaded in a syringe with a coaxial single spinneret consisting of an inner nozzle for the precursor solution and an outer nozzle for the high-speed air used. Blow-spinning is divided into solution spinning and melt spinning. Solution spinning can be divided into dry spinning and wet spinning according to the direction of the spinning solution from the nozzle. For instance, a robust photocatalytic composite SiC@SiO_2_/carbon nanofibre mat is prepared via facile blow-spinning (Figure 2). The spinning dope was fed into a 0.5 mm diameter needle and then stretched by airflow with 0.12 MPa pressure. The synthesized composite exhibited excellent photodegradation of dyes that showed good recycling performance with a dye degradation above 88–95% after 5 cycles, thanks to the utilization of PAN-based carbon nanofibre mats and high chemical stability under both alkaline and acidic environment environments (Chen Y. et al., 2019).

### Electrospinning

Electrospinning is a simple and versatile method that can be used to synthesize nanofibres with high porosity, high specific surface area, and excellent mechanical strength. It is a top-down technology where there is a high voltage field between the precursor solution and a conductive substrate. It can make the drop of the solution with the charge in the electric field overcome the surface tension to emit Taylor cone to fabricate ultrafine nanofibres. A porous, free-standing carbon electrode with a high specific surface area was prepared by electrostatic spinning. Jennifer S. Atchison and his group obtained sub-micrometer-sized fibers that were homogenously composed of nanodomains of metal carbides such as ZrC/C, TiC/C, NbC/C. The range of fiber diameters and the specific surface area is between 294 - 108 nm and 224 m^2^ g^−1^ for ZrC/C, 122 - 28 nm and 450 m^2^ g^−1^ for TiC/C, as well as 65 - 36 nm and 121 m^2^g^−1^ for NbC/C (Atchison et al., 2015). These metal carbide/carbon nanocomposite fibers were acquired in the form of freestanding non-woven textiles that could serve as an ideal precursor for synthesizing highly porous carbide-derived carbon electrodes for electrochemical applications. However, it is worth noting that the instability of the precursor with a relatively high concentration of inorganic salts often arises during the electrospinning process because the electrical conductivity of the precursor rises with the increase of inorganic salts content and the electrospinning has to bear the risk of high voltage, low productivity, complex operating conditions which limits the application of this technology in large-scale nanofibre production.

### Centrifugal Spinning

**Figure 3:**
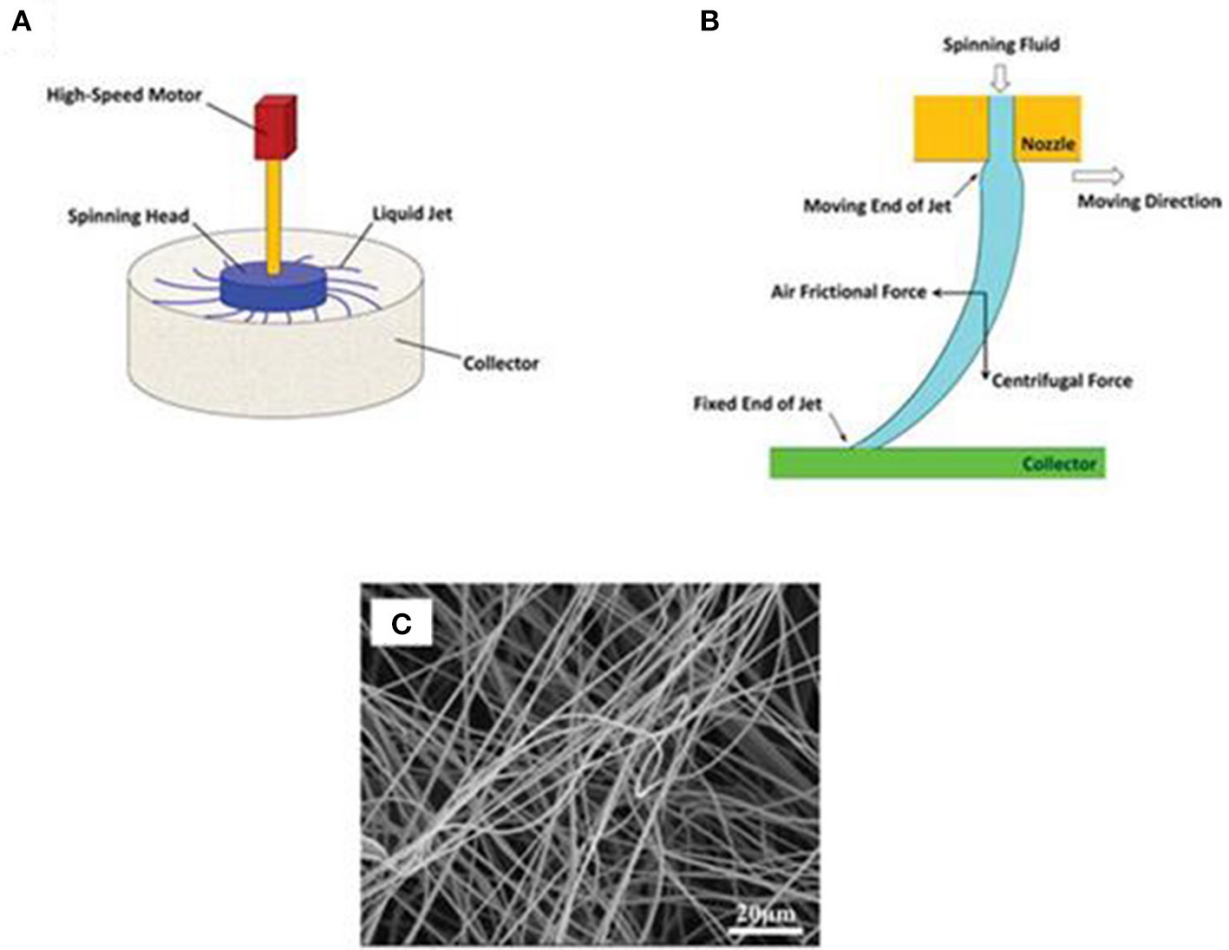
**(A)** Schematic of a basic bench-top centrifugal spinning setup. **(B)** The path of a liquid jet ejected from the nozzle tip during centrifugal spinning. **(C)** SEM image of PAN nanofibers prepared by centrifugal spinning. Reproduced with permission from Zhang and Lu (2014).

As a new spinning method, which is mainly used in the spinning of glass fiber, phenolic and general grade pitch CFs, centrifugal spinning can solve the shortage of electrostatic spinning to some extent. It uses centrifugal force instead of the electrostatic to accomplish the formation of precursor fibers. Therefore, regardless of whether the polymer has good conductivity, it no longer needs the restriction of high voltage, which can sharply reduce the cost of spinning. Moreover, one of the important characteristics is its high production rate (Zhang and Lu, 2014; Song et al., 2019) (Figure 3). The formation mechanism of Ethyl cellulose (EC)/PVP fiber between centrifugal spinning and electrospinning under the same solution and ambient conditions (EC/PVP = 90/10%) was discussed. Through a binary solvent system of ethanol and water (ethanol/water = 70/30%), the micro-porous and nano-porous structures are fabricated by centrifugal spinning with the rotational speed of the spinneret controlled at 3,500 rpm. The wonderful performances of centrifugal spinning may prove that it is not only a novel technique but also a viable alternative for the production of long continuous, non-woven mats of nanofibres at a considerable higher yield.

### Others

Compared with the preparation of single-component fibers, composite fibers have more extensive applications due to their various components and structures. The preparation of composite fibers is mainly based on coaxial spinning by using two coaxial spinnerets with different inner diameters. This technique can solve the problem of the spinning solution that needs to be a uniform system when preparing composite fibers or the fibers with special structures (e.g., hollow fibers) and it can also properly combine the efficiency and advantages of two kinds of materials to make them deliver excellent properties that they never had when they are used alone (Zhang et al., 2020).

## Carbonization

The main purpose of the carbonization process is to remove most of the oxygen, nitrogen, hydrogen and other elements in precursor fibers by thermal decomposition in order to increase the carbon content (reach at least 90%) and make the coupling reaction between adjacent carbon chains to occur. The whole carbonization process can be summarized into three parts: pre-oxidation, low-temperature carbonization, and high-temperature carbonization (Karacan and Erzurumluoglu, 2015; Byrne et al., 2016; Hameed et al., 2016). The pre-oxidation takes place in an oxygen environment to modify more oxygen-containing functional groups on the surface of precursor fibers and the temperature is controlled within the range of 200–300°C by utilizing oven or muffle furnace as the heating equipment. Through the process, a series of reactions such as dehydrogenation, cyclisation, aromatisation and crosslinking occurred for transforming the linear molecular chain to a conjugated ladder structure thus improving the thermal stability of the precursor fiber as well as the crystallinity of CFs will increase via forming C=C bonding. The whole carbonization process takes place in the nitrogen environment under a temperature of at least 800°C by furnace or tube-furnace to increase the proportion of carbon element. Compared with untreated CFs, by increasing boron content in the fiber can improve the tensile strength and modulus by 16 and 26%, respectively. Because the substitutional and interstitial diffusion of boron can remove structural defects and relax the distortions in the CF structure to enhance its mechanical properties (Diani et al., 2006; Qin et al., 2012). The stable heating rate during carbonization directly affects the performance of CFs. Too high heating rate will introduce defects, while too low heating rate will waste more nitrogen in the early stage. To avoid thermal shock on CFs, the low-temperature carbonization process mentioned above is very necessary.

## Functional CFs

CFs have been widely studied as nano-materials because of their outstanding chemical and physical properties (Wei and Qu, 2012). However, with the development of technology, in order to meet the research of some specific performance nanomaterials, more and more functional materials are compounded onto the surface of CFs, which significantly improve the properties of composites, such as precious metal nanomaterials, polymers, metal oxides, MOFs and so on. Here, we review the strategies of these common functional CFs.

### Noble Metal Functionalised CFs

**Figure 4:**
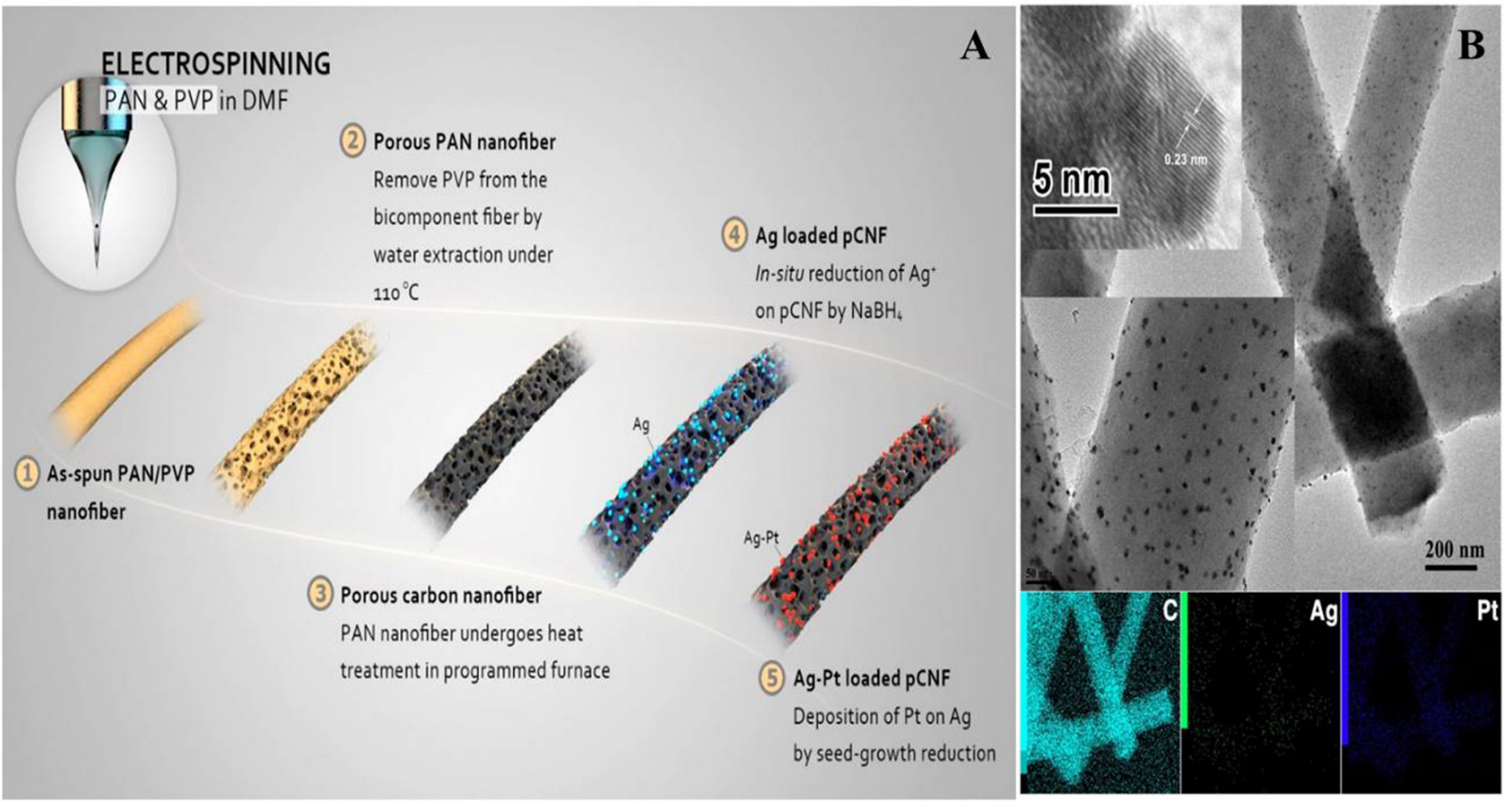
**(A)** Schematic illustration of the preparation of Ag–Pt/pCNFs. **(B)** TEM images of Ag–Pt/pCNFs and the corresponding elemental mappings. Reproduced with permission from Huang et al. (2014).

Precious metal nanomaterials have empty d-electron orbitals, small energy level gaps, easy to form coordination bonds, and easy to adsorb and desorb groups on their surface, so they form intermediate active compounds easily, thus allowing them to have high catalytic activity. In recent years, precious metal nanomaterials have been often used as nanomaterials to modify the surface of CFs because of their excellent stability, good electrical conductivity and high biocompatibility. At the same time, the production and modification of metal nanoparticles are relatively simple. In order to increase the specific surface area and enhance the electrochemical performance of CFs, gold nanoparticles were used to etch the surface of CFs. The results show that etching not only reduces the diameter of CFs, but also affects the morphology and roughness of CFs surface by producing defects or porous structures. At the same time, it is also found that this etching method will not destroy the sp^2^ bonding of graphite, but may focus on grain boundaries or defects (Long et al., 2017). CFs modified by Au NPs are also used to detect cancer cells. Through the preparation of CFs microelectrode with hierarchical nanostructure of Au-MnO_2_/GO/CF, the grapheme-based composite enhanced the specific surface area of CFs and improved the electrical conductivity of the material, and then the MnO_2_ on CFs formed porous nanostructures, it provides an excellent matrix for the growth of Au NPs. The results show that the prepared microelectrode can realize real-time, rapid and sensitive detection of hydrogen peroxide secreted by human cervical cancer cells (Abdurhman et al., 2015). At the same time, some researchers directly electrodeposited gold nanoparticles on the surface of CFs to monitor the release of dopamine and to determine the intracellular exocytosis of rats (Barlow et al., 2018). At present, there are few reports on electrochemical detection of dopamine with bimetallic nanomaterials. Based on this, researchers have prepared Ag-Pt bimetallic nanomaterials modified CFs microelectrodes to achieve rapid and sensitive detection of dopamine (Figure 4). The results show that the sensor has the advantages of fast current response, high sensitivity, wide detection range and low detection limit, and can effectively eliminate the effect of interfering substances on the detection results of dopamine (Huang et al., 2014). In conclusion, precious metal composite CFs nanomaterials show the synergistic effect of various components, which further improve the properties of the composites, especially in the application of batteries and capacitors. In the future, precious metals will remain competitive in the application of functional CFs.

### Metal Oxide Functionalised CFs

As the first choice of catalysts, metal oxide nanomaterials are widely used in the catalytic reaction of oxidation-reduction mechanism (Navrotsky et al., 2010), including the fabrication of various sensors and anode materials for batteries, etc., and the combination of metal oxides and CFs is also a common strategy of functionalized CFs. The researchers synthesized a humidity sensor based on bismuth ferrite nanoparticles BiFeO_3_ (BFO), and successfully prepared a high-performance humidity sensor by combining BFO with CFs by hydrothermal method. Compared with BFO as humidity sensitive units, composite nanomaterials exhibit high sensitivity, low hysteresis and excellent stability, which proves the potential of BFO in humidity sensing (Douani et al., 2020). With the fine requirements of blasting, the ignition device of detonation has become the focus of researchers' exploration, in which the energy conversion element is the core of the research hotspot. The preparation of high-quality energy conversion elements can enhance the ignition efficiency, shorten the ignition delay and improve the success rate of detonation. Therefore, taking advantage of the good electrical conductivity and easy surface modification of CFs, the researchers prepared a new type of ignition device by compounding Al/BiO_3_ nanomaterials on the surface of CFs, in which Al/BiO_3_ can improve the detonation ability and reliability. As the energy conversion element of the ignition device, this is a new application field of CFs materials, and it also develops a new research idea for the ignition device (Yi et al., 2020).

Transition metal oxides are widely used as electrode materials for energy storage devices because of their easy availability and high capacitance. Combining transition metal oxides with CFs has been proved to be an effective method to improve the specific capacitance and energy density of devices. It will still be a hotspot in the research of battery devices in the future (Ma et al., 2017).

### Polymers Modified CFs

CFs composites have excellent tensile properties and stiffness, as well as light and thin, good heat resistance and other advantages, so they are ideal structural materials and are widely used in medical, construction, transportation, aerospace and other fields (Li et al., 2015). Among them, the degree of interfacial adhesion between CFs and matrix is the key to determine the properties and structure of CFs composites (Zhang et al., 2018a). Polymer molecules are often reported to be used to modify CFs to enhance their interfacial stickiness. Based on this, we summarize the research and application of some polymer matrix modified CFs in recent years. They compounded the polydopamine-nickel modified CFs material with rigid polyurethane (RPU) and studied the mechanical and electrochemical properties of the composite (Huang et al., 2020). Compared with the original CFs-RPU composites, the strength, toughness and electrical properties of the modified CFs composites are significantly improved due to the chemical cross-linking between CFs and RPU interface. A green functionalisation method for the modification of CFs in water with polyoxypropylene diamine (D400) as the coupling agent and curing agent was reported (Wang et al., 2017). Through the study of the microstucture and mechanical properties of the modified composites, it was found that D400 not only did not change the surface structure of CFs, but enhanced the polarity, lubricity and roughness of CFs surface.

Polymer reinforced CFs composites have attracted wide attention from researchers because it combines the good electrical conductivity and mechanical properties of CFs and the excellent interfacial adhesion and thermoplasticity of polymers (Liu and Kumar, 2014; Wen et al., 2019). It can not only be used for the preparation of microdevices, such as sensors, energy storage devices but also in aerospace, automotive industry and other heavy industry assembly. CFs reinforced composites still have broad application prospects.

### Metal-Organic Frameworks (MOFs) Functionalised CFs

**Figure 5:**
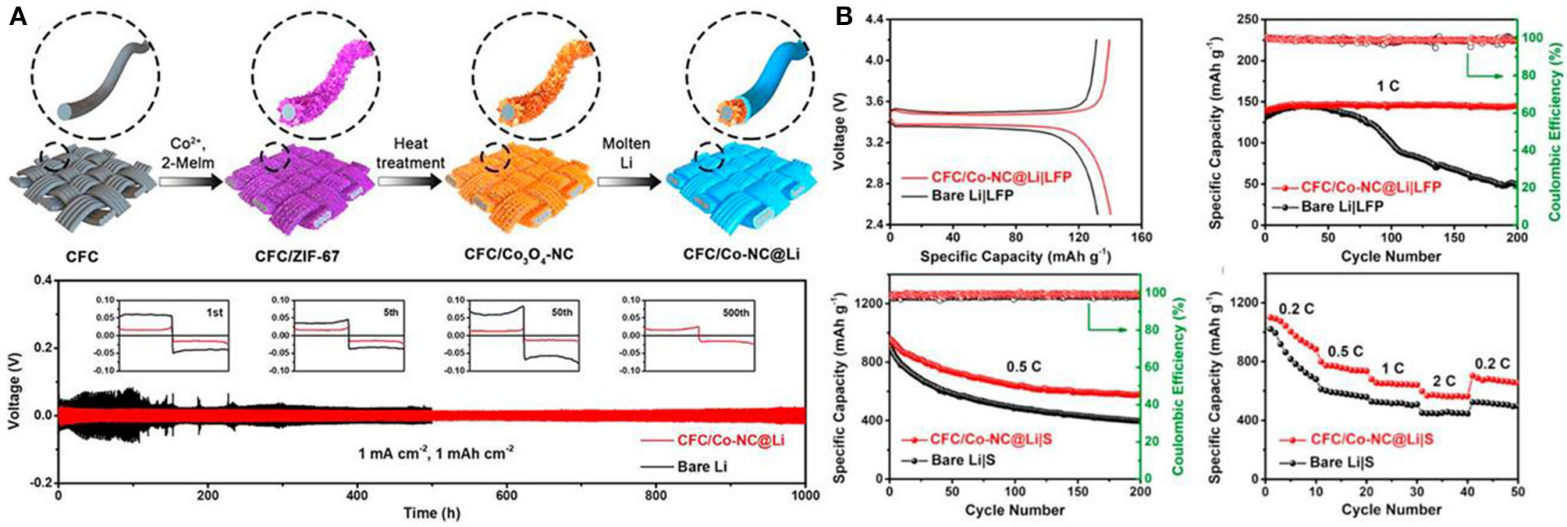
**(A)** Schematic illustration for the fabrication of CFC/Co-NC@Li anode. **(B)** Electrochemical performance of composite materials assembled into full batteries. Reproduced with permission from Jiang et al. (2019).

MOFs are new organic porous materials, also known as porous coordination polymers (PCPs). Generally speaking, they are composed of two main components: metal ions or clusters and organic ligands, both of which are mainly assembled by clear coordination bonds. However, the further application of pure MOFs is limited because of their unique shape, limited function and unsatisfactory performance. In recent years, MOFs composites have become a new research hotspot. For example, inorganic materials, carbon materials, metal nanocrystals, polymers and biomolecules have been proved to be able to combine with MOFs to form new multifunctional composites. MOFs composites are widely used in sensors, batteries, supercapacitors, gas storage and separation, catalysis and so on. In this part, we summarize the applications and challenges of MOFs-CFs composites in these fields in recent years (Li et al., 2017; Jiao et al., 2019; Meng et al., 2020). Lithium-ion batteries and sodium-ion batteries have been widely used in energy storage systems, in which the selection of electrode materials is always the main factor affecting the performance of batteries. CFs have been widely studied as one of the battery anode materials, but their low reversibility in battery manufacturing hinders their further application. For this reason, MOF-derived, Co_3_O_4_-intercalated and nitrogen-doped porous carbon nanoarrays were prepared on CFs sheets (CFC/Co_3_O_4_-NC) as anode materials for lithium-ion batteries for the first time (Jiang et al., 2019). Then the matrix is combined with the molten Li, and the molten Li reacts with the matrix to obtain a composite anode (CFC/Co–NC@Li), which can effectively slow down the volume change and inhibit the growth of Li dendrites (Figure 5). Repeated stripping/plating Li 500 cycles (1,000 h) at low potential (18 mV) still shows excellent stability and long service life.

In addition, MOF modified CFs composites are also used to improve interfacial properties and efficient oxygen evolution reaction. MOFs show remarkable advantages in improving the interfacial properties of composites (Zhu and Xu, 2014). Researchers hope to improve the interfacial properties of CFs/epoxy composites by growing nano-flake MOFs on the surface of CFs (Li et al., 2020). CFs modified by nano-flake MOFs have a uniform surface structure, and the interfacial shear strength and surface energy increase by 70.30 and 69.75%, respectively, under the action of MOF. As a new kind of porous crystal materials, MOFs have been successfully prepared. However, because of their inherent poor chemical stability and weak conductivity, they are often used as precursors or templates to synthesize various carbon-based structures (Choi et al., 2011). It is still a challenge to build unique MOF-based composites and improve their properties in the future.

### Other Materials Functionalised CFs

In addition to the above-modified materials, nanomaterials such as graphene oxide and carbon nanotubes are also used to functionalise CFs. CFs composites containing carbon nanotubes and graphene have significantly improved mechanical, electrical and thermal properties (Chou et al., 2010; Kong et al., 2013; Rahmanian et al., 2013). By coating the carboxylated carbon nanotubes on the CFs microelectrode, a micron biological electrode with high specific surface area that can be used as the carrier of immobilized enzyme was prepared (Wen et al., 2011). The results show that the CFs electrode modified by carbon nanotubes can make the current density of the electrode show a quantifiable and observable increase, compared with the bare CFs electrode, the modified electrode increases by 2,000 times. At the same time, it is also found that due to the hydrophilicity of carboxylated carbon nanotubes, the biocatalyst precursor enters into the porous structure to form carbon nanotube-hydrogel composites, which can increase the concentration of active media and enzymes. The current density of the modified glucose oxidase electrode can be increased by 6.4 times to 16.63 mA cm^−2^. This study laid a foundation for the preparation and application of bioelectrode and biofuel cell supported by CFs. Carbon nanotubes have greatly improved the sensitivity of the sensor, and this sensor has been successfully applied to the detection of NO release from human venous endothelial cells. Graphene oxide has the characteristics of micron size, high aspect ratio and two-dimensional flake geometry, which can effectively deflect cracks when bending/shearing at the interface of composites (Xu and Buehler, 2010). Sizing agents containing different concentrations of graphene oxide to modify the interface of CFs was reported (Zhang et al., 2012; Jiang et al., 2017). Through the study and analysis of the morphology, interfacial shear strength and thermomechanical properties of CFs composites, it is proved that the mechanical properties of CFs/epoxy composites can be significantly improved by introducing graphene on the surface of CFs/epoxy composites. The composites with improved mechanical properties may show great application potential in the automotive industry and aerospace industry.

## Application of Biosensor Based on CFs

As an important technology, an electrochemical biosensor has the advantages of simple operation, rapid analysis, high selectivity, and low cost etc. In recent years, with the rapid development of sensors, the requirements for sensors are increasing. (e.g., high sensitivity, low detection limit, excellent biocompatibility and stability etc.). It has been paid high attention and widely used in environmental detection, food industry, fermentation industry, biomedical research, and other fields. Due to the outstanding properties of CFs such as low relative density, good mechanical strength, high-temperature resistance and their structure can be adjusted into various geometric shapes according to the different needs. With the rapid rise of flexible electrodes and wearable electronic products, biosensors are not limited to the laboratory level. Researchers integrate CFs materials into flexible devices and wearable electronic devices to prepare a new type of miniaturized and portable biosensors. Now, biosensors based on flexible electrodes and wearable devices of CFs nanomaterials have been widely used in environmental analysis, food safety, biomedicine and human health monitoring.

### Conventional Electrochemical Biosensor Based on CFs

The porosity of CFs based electrodes is conducive to the penetration of electrolyte and the diffusion of ions and the continuous conducting network can achieve the rapid transfer of charge between active substances and metal ions. CFs have become a biosensing platform for the detection of biomolecules because of their good biocompatibility, excellent electrical conductivity and robust mechanical properties. Furthermore, thanks to their unique electrochemical properties caused by small size which possess micron size in one dimension, microelectrodes have attracted considerable interests in electrochemical analysis. For instance, the core-shell structure of two-dimension VS_2_@VC@N-doped carbon sheets decorated by ultrafine Pd nanoparticles vertically grown on CFs by a modified template-free hydrothermal method, which assembled into a unique 3D rosette-like array was used to fabricate an H_2_O_2_ electrochemical microsensor (Yuan et al., 2020). This biosensor showed excellent electron transfer ability, electro-catalytic activity, stability and biocompatibility because of the unique rosette-like array structure. It could be used for real-time *in situ* electrochemical detection of H_2_O_2_ in live cancer cells and cancer tissue, exhibiting a high sensitivity of 152.7 μA cm^−2^ mM^−1^, and a detection limit (LOD) of 50 nM (a signal-to-noise ratio of 3:1) as well as great reproducibility and anti-interference ability. In addition to detecting H_2_O_2_, the electrode fabricated (Wu et al., 2019) consists of nitrogen-doped cotton carbon fibers (NCFs) modified with silver nanoparticles by eletrodeposition has been proposed as a biosensor with excellent catalytic capability for superoxide anion release from cells either under normal or under oxidative stress conditions. The electrochemical sensor operates at a low potential of −0.5 V (vs. SCE), displayed a marvelously wide range that covers 10 orders of magnitude, as well as the detection limit is 2.32 ± 0.07 fM. NCFs were synthesized by a two-step process. The NCFs were prepared via drying in a vacuum oven at 80°C for 24 h and then directly carbonized at 800°C below a nitrogen atmosphere to form nitrogen-doped cotton CFs. The silver nanoparticles were grown on the surface of the modified CFs electrode using a one-step electrodeposition technique. CFs-based biosensors are also widely used to monitor human physiological indexes and cellular active components. Cortisol is involved in the regulation of a variety of physiological activities and is considered to be a key factor in stress response and bio-psychology. Researchers (Loaiza et al., 2015) prepared a lactate biosensor based on graphitised carbon nanofibres to detect lactate in wines and ciders. Graphitised carbon nanofibres supported Pt NPs composites (Pt NPs/GCNF) were prepared by chemical reduction of Pt precursors on the surface of GCNF for lactic acid sensing and the lactate oxidase (Lox) was modified by covalent immobilization onto the Pt NPs/GCNF surface using polyethyleneimine (PET) and glutaraldehyde (GA). The lactic acid sensor shows excellent reproducibility (RSD 4.9%, n=10) and sensitivity (41.302 ± 546) μA/M cm^2^, with a good detection limit (6.9 μM). At the same time, it is proved that the activity of the sensor can be preserved about 95% under the storage condition of −20°C, which greatly improves the accuracy and sensitivity of lactic acid detection in beverages.

### Flexible or Wearable Biosensor Based on CFs

Flexible electronic devices and wearable smart devices have developed rapidly in recent years. Instead of reducing the detection accuracy and sensitivity, they make the equipment miniaturized, portable and intelligent. Therefore, flexible devices and wearable devices based on CFs biosensors still have broad prospects for development in the future.

**Figure 6:**
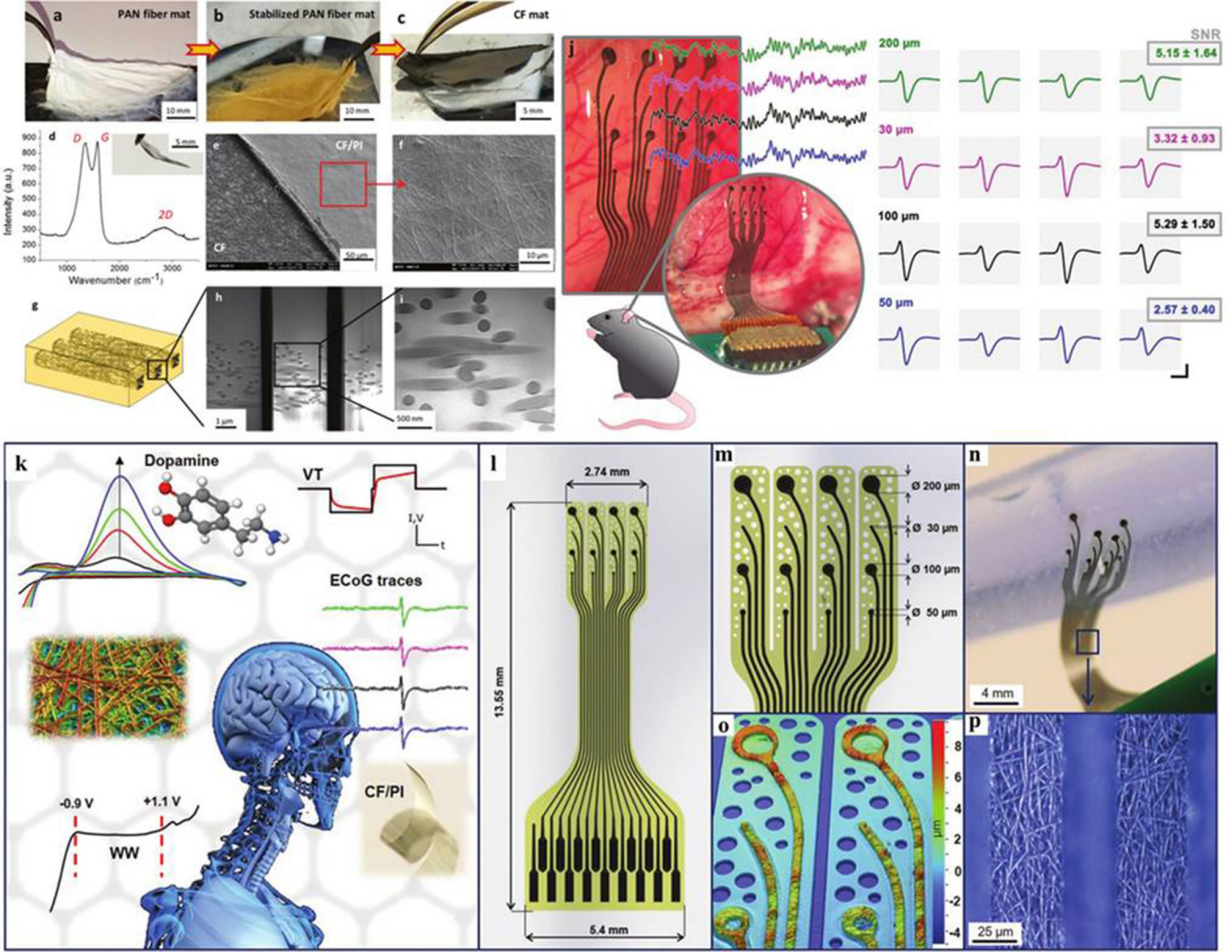
**(a–i)** Fabrication and characterization CF mats. **(j)** Applications and characterization of CF-based flexible device. **(k–p)** *In vivo* characterization of CF-based flexible neural implants. Reproduced with permission from Vomero et al. (2019).

The monitoring of brain activity has practical significance for biological physiological health signals. Researchers (Vomero et al., 2019) reported that a flexible biosensor probe based on CFs was implanted into mouse brain tissue (Figure 6). A micromachining technology for embedding flexible, cloth-like and polymer-derived CFs pads in polyimide by selective reactive ion etching is introduced. The whole electrocorticography (ECoG) electrode array is seamlessly composed of a single CF pad, avoiding any joint and metal interconnection. In the process of wafer fabrication, the plane resolution of CFs structure is reduced to 12.5 μm and the height is 3 μm. The prepared superflexible neural device has good electrochemical stability and excellent mechanical properties *in vitro*, and shows good recording performance after implantation *in vivo*. Although this study focuses on the preparation of ECoG electrode, the preparation technique of metal-free implantable probe based on polyimide/CF can also be used in other biomedical monitoring and sensing platforms. Similarly, to monitor the H_2_O_2_ secreted by cancer cells *in vivo* in real-time, researchers (Zhang et al., 2018b) reported a preparation strategy of hybrid flexible microelectrode based on CF through hydrothermal synthesis, which uses CF coated gold nanoparticles modified nitrogen hybrid carbon nanotube arrays (NCNATs). CF is an ideal substrate for *in situ* monitoring because of its nanometer scale and excellent mechanical properties. NCNATs grown on CFs significantly enhanced the electrochemically active surface area and enriched the surface active sites. The gold nanoparticles uniformly distributed on NCNATs provide a guarantee for the electrochemical detection of H_2_O_2_. In the selective detection of H_2_O_2_, the detection limit of the composite microelectrode is 50 nM when the signal-to-noise ratio is 3:1, the linear range is up to 4.3 mM, and the sensitivity is as high as 142 μA cm^−2^mM^−1^. The composite flexible microelectrode for real-time tracking and monitoring of H_2_O_2_ secreted by cancer cells can promote the development of detection and management of early diseases. At present, in the field of biosensors, there are still some challenges in transforming sensing materials into wearable devices. Researchers (Aaron et al., 2018) reported a simple synthesis strategy of biosensors based on CFs to detect peroxides. It provides an effective solution for the application of biosensors in the field of wearable devices. In terms of details, palladium nanostructures were deposited on the surface of CFs by electrodeposition. Through electrodeposition, palladium nanostructures formed nanoneedles and nanorods that were vertically attached to the surface of CFs. Through the detection of peroxide, the flexible electrode shows high sensitivity of 388 μA mM^−1^ cm^−2^. This simple preparation strategy provides a reference idea for the development of wearable biosensors.

## Summary and Outlook

In summary, we review the preparation methods and functional modification of CFs and the application of CFs-based composites in biosensors. In the preparation process of CFs, we introduce the preparation process in detail. Firstly, the raw materials for the preparation of CFs are classified, including i) Natural polymers; ii) Synthetic polymers. In the part of raw material pretreatment and spinning, we list several common spinning methods, which are i) Blow spinning; ii) Electrospinning; iii) Centrifugal spinning; iv) Others. The last step in the preparation of CFs materials is the carbonization process, which is mainly divided into three steps, namely i) pre-oxidation (to improve the thermal stability and crystallinity); ii) low-temperature carbonization; iii) high-temperature carbonization (to enhance the mechanical properties). Next is the review of the functionalisation of CFs. In this part, we generalize four common functionalisation methods and other modified materials, which are i) Precious metal functionalised CFs; ii) Metal oxide functionalized CFs; iii) High molecular polymer-modified CFs; iv) Metal-organic frameworks (MOFs) functionalised CFs; v) Other materials functionalised CFs. Through the functional modification of CFs, the unique properties of each part of the materials have a synergistic effect and show excellent comprehensive properties. As a result, CFs-based composites show a broad application prospect in the fields of sensing, electric energy storage equipment, industrial manufacturing and so on. In the last part, we introduce in detail the application of CFs-based composites in flexible or wearable biosensors.

As we all know, CF, as a very advanced and omnipotent nano-material, has been proved to have a wide range of application prospects. At present, the preparation and production process of CFs has been quite advanced, and its properties have been explored a little bit. In the future research direction, CFs will still be very popular nano-based materials. When studying the application of CFs in flexible biosensors, we found that the application of CFs in flexible biosensor may be limited due to the complexity and cost in the preparation process of composite materials. On the contrary, CFs are the most preferred materials in the fields of electrical energy storage and industry, because of their excellent mechanical properties. At the same time, it is not uncommon for CFs-based biosensor probes to be implanted in organisms, which shows that CFs-based nanoprobes also have a broad application space in the field of biomedicine (Saito et al., 2011). Therefore, it is still worthy of our attention to explore how to create a balance between the application of CFs in flexible biosensors and the preparation of composites.

## Author Contributions

ZW and MF contributed equally to the article and they wrote the article together. QY and CF are responsible for providing the required materials. YH and ZM provided ideas and support for the whole review. All authors contributed to the article and approved the submitted version.

## Conflict of Interest

The authors declare that the research was conducted in the absence of any commercial or financial relationships that could be construed as a potential conflict of interest.
